# Attention-based speech feature transfer between speakers

**DOI:** 10.3389/frai.2024.1259641

**Published:** 2024-02-26

**Authors:** Hangbok Lee, Minjae Cho, Hyuk-Yoon Kwon

**Affiliations:** Department of Industrial Engineering, Seoul National University of Science and Technology, Seoul, Republic of Korea

**Keywords:** speech synthesis, attention mechanism, speech features, feature transfer, speech similarity

## Abstract

In this study, we propose a simple yet effective method for incorporating the source speaker's characteristics in the target speaker's speech. This allows our model to generate the speech of the target speaker with the style of the source speaker. To achieve this, we focus on the attention model within the speech synthesis model, which learns various speaker features such as spectrogram, pitch, intensity, formant, pulse, and voice breaks. The model is trained separately using datasets specific to the source and target speakers. Subsequently, we replace the attention weights learned from the source speaker's dataset with the attention weights from the target speaker's model. Finally, by providing new input texts to the target model, we generate the speech of the target speaker with the styles of the source speaker. We validate the effectiveness of our model through similarity analysis utilizing five evaluation metrics and showcase real-world examples.

## 1 Introduction

Speech synthesis has been extensively studied, particularly in the context of text-to-speech (TTS) systems (Ren et al., 2019; Kaur and Singh, 2022; Kumar et al., 2023). In the context of TTS technology, the traditional process involved three steps: (1) tokenizing the input text, (2) conducting rhyme analysis, and (3) searching and concatenating segmented speech units to convert the text into speech. However, with recent advancements in the field of speech synthesis, deep learning-based approaches have gained significant traction. This means that instead of relying on the conventional approach comprising multiple subprocesses, there has been a notable shift toward the development of end-to-end TTS technology (Ren et al., 2020), which is supported by trained models.

In this study, we focus on end-to-end speech synthesis for our speech feature transfer between speakers. Recently, although more advanced models have been proposed such as Naturalspeech (Tan et al., 2022), Delightfultts 2 (Liu et al., 2022), and Wave-tacotron (Weiss et al., 2021), we propose our method based on Tacotron because Tacotron (Wang et al., 2017) is the representative end-to-end acoustic model and is the most widely used acoustic model (Mu et al., 2021). However, we note that our approach for speech feature transfer can be applied to any model along with an attention module.

Google's Tacotron (Wang et al., 2017) takes text as input and produces low-level spectrograms as output. Unlike traditional speech synthesis models trained at the word level, Tacotron operates at a more detailed phoneme level, enabling it to generate natural pronunciations through phoneme combinations. The Tacotron model comprises four essential modules: (1) encoder, (2) decoder, (3) attention module, and (4) vocoder. The encoder module transforms the text input into continuous vector values through one-hot encoding. The decoder module takes the processed vector values from the encoder and generates spectrograms, which represent the speech output. Spectrograms visually depict the spectrum of frequencies over time and serve as the intermediary step in converting numerical values into speech. The decoder consists of multiple recurrent neural networks (RNNs) that predict future words based on the relationship between previous and current words. Through these RNNs, the decoder generates the next spectrogram based on the information from the preceding spectrogram. The attention module serves as the vital link between the encoder and decoder, facilitating the creation of natural-sounding utterances. It learns the mapping relationship between the output and input, determining where to focus attention in order to generate speech. This mechanism enables the model to learn speaker-specific intonation, speech rate, and style from a diverse range of datasets. The vocoder module takes the spectrograms generated by the decoder and converts them into audible speech. It performs the final step of synthesizing speech from the spectrogram representation. Tacotron utilizes these interconnected modules to synthesize speech. Through training on various datasets, the model learns the speaker's intonation, speech rate, and other speech characteristics, ultimately enabling the synthesis of high-quality and speaker-specific speech.

In this study, we propose a simple yet effective method for incorporating the source speaker's characteristics in the target speaker's speech. This allows our model to generate the speech of the target speaker with the style of the source speaker. For instance, if we wish to generate text from the target speaker at the speech speed of the source speaker, our model can facilitate this capability. To achieve this, we focus on the attention model within the speech synthesis model, which learns various speaker features such as spectrogram, pitch, intensity, formant, pulse, and voice breaks (Yasuda et al., 2019). We employ Google's Tacotron (Wang et al., 2017) as the foundational model for speech synthesis. The model is trained separately using datasets specific to the source and target speakers. Subsequently, we replace the attention weights learned from the source speaker's dataset with the attention weights from the target speaker's model. Finally, by providing new input texts to the target model, we generate the speech of the target speaker with the styles of the source speaker. We validate the effectiveness of our model through similarity analysis utilizing five evaluation metrics and showcase real-world examples.

## 2 Method

To incorporate the features of the source speaker into the target speaker's synthesis model, we follow five main steps: (1) data preparation, (2) data preprocessing, (3) model training, (4) feature transfer, and (5) speech synthesis.

### 2.1 Data preparation

To train Tacotron with a single speaker, a large supervised dataset specific to that speaker is essential. In this study, we utilized three different datasets to gather an extensive amount of data. (1) We collected six audiobooks by Benedict Cumberbatch, namely “Artists in Crime,” “Casanova,” “Death in a White Tie,” “Metamorphosis,” “Scale of Justice,” and “Sherlock Holmes.” The combined duration of these audiobook files amounts to ~13 h. (2) We also incorporated the LJ Speech Dataset, which is an open dataset widely used for speech synthesis tasks^1^. (3) We utilized World English Bible (WEB) Speech Dataset, which is another open dataset available on Kaggle. This dataset contains audio recordings of the World English Bible^2^.

### 2.2 Data preprocessing

The collected audio dataset cannot be directly used for training due to the limitations on the length of audio files that can be processed by speech synthesis deep learning models. To address this, the original audio files were subjected to an editing process since they needed to fit the specific batch size set in the model, which in this case is 12.5 s (the default size of Tacotron). In order to avoid negative impacts on training, all audio files with a duration of <2 s were removed. Consequently, ~11 h of training data were retained from the original 13-h dataset in the case of Benedict Cumberbatch's audiobooks.

The next crucial step is the labeling process for these audio files. To effectively handle large-scale datasets, the Google Speech-to-Text API was utilized. While there were instances where slightly distorted text was returned for similar pronunciations, it was observed that Google's Tacotron model has an internal algorithm that splits input sentences into phoneme units. Therefore, even if the returned text was somewhat distorted, it was deemed that this would not significantly affect the training process as long as the pronunciation remained similar.

### 2.3 Model training

In this study, we constructed a separate Tacotron model for each speaker under investigation. Since we are working with three speakers in this paper, we developed a total of three Tacotron models. These models, specific to each speaker, are utilized for the purpose of speech synthesis.

For training each Tacotron model, we used a set of hyperparameters, as presented in Table 1. While primarily following the hyperparameters recommended by Tacotron, we fine-tuned them to suit our specific dataset. To find more suitable settings, we focused on the adjustment of dropout probability (i.e., 0.5–0.8), attention size (i.e., 128–512), and reduction factor (i.e., 2–5) and found the current setting as the most suitable one.

**Table 1:** The used hyperparameters.

**Spectral analysis**	**Pre-emphasis: 0.97; frame length: 50 ms; frame shift: 12.5 ms**
Character embedding	256-D
Encoder CBHG	Conv1D bank: *K*=16, conv-*k*-128-ReLU Max pooling: stride = 1, width = 2 Conv1D projections: conv-3-128-ReLU → conv-3-128-Linear Highway net: 4 layers of FC-128-ReLU Bidirectional GRU: 128 cells
Encoder pre-net	FC-256-ReLU → Dropout(0.5) → FC-128-ReLU → Dropout(0.5)
Decoder pre-net	FC-256-ReLU → Dropout(0.5) → FC-128-ReLU → Dropout(0.5)
Decoder RNN	2-layer residual GRU (256 cells)
Attention RNN	1-layer GRU (256 cells)
Post-processing net CBHG	Conv1D bank: *K*=8, conv-*k*-128-ReLU Max pooling: stride = 1, width = 2 Conv1D projections: conv-3-256-ReLU → conv-3-80-Linear Highway net: 4 layers of FC-128-ReLU Bidirectional GRU: 128 cells
Reduction factor	5

“conv-*k*-*c*-ReLU” denotes 1-D convolution with width *k* and *c* output channels with ReLU activation. FC, fully-connected.

### 2.4 Feature transfer

To inject the source features into the target speaker, the attention module's output values generated by the Tacotron model trained on the source dataset are extracted and utilized in conjunction with the Tacotron model trained on the target dataset. When generating speech using the target model, the attention module of the source model is employed to focus on the speech characteristics present in the source dataset. The extracted attention values serve as a guide for the target model, enabling it to incorporate the speech features of the source dataset into the synthesis process. By leveraging the attention mechanism and incorporating the source dataset's speech characteristics, the target model produces synthesized speech that retains the desired features and qualities of the source speaker.

### 2.5 Speech synthesis

By utilizing the target speaker's model with replaced attention weights learned from the source speaker's datasets, we proceed with the speech synthesis process. This synthesis generates speech from the target speaker that incorporates the features derived from the source speaker's datasets. Consequently, when inferring new input texts using the modified target model, the synthesized speech reflects the source speaker's voice and style.

## 3 Results

### 3.1 Qualitative analysis

**Figure 1:**
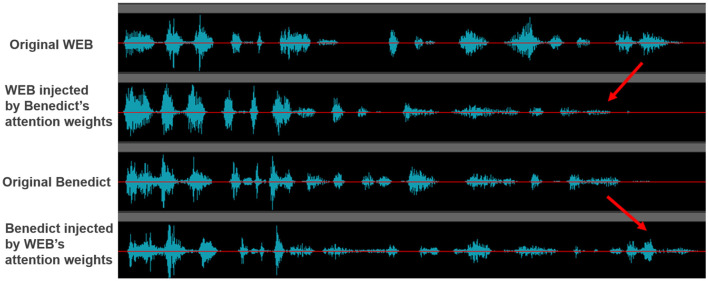
The effects of transferring the learned attention weights to the speech strength.

To validate the effectiveness of our transfer mechanism, we compare the following two major speech features: (1) speech strength and (2) speech speed. Figure 1 shows the change in the speech strength with our method. We note that WEB's speech, which injects Benedict's attention weights, becomes more similar to Benedict's one. Similarly, Benedict's speech, which injects WEB's attention weights, becomes more similar to WEB's one^3^.

**Figure 2:**
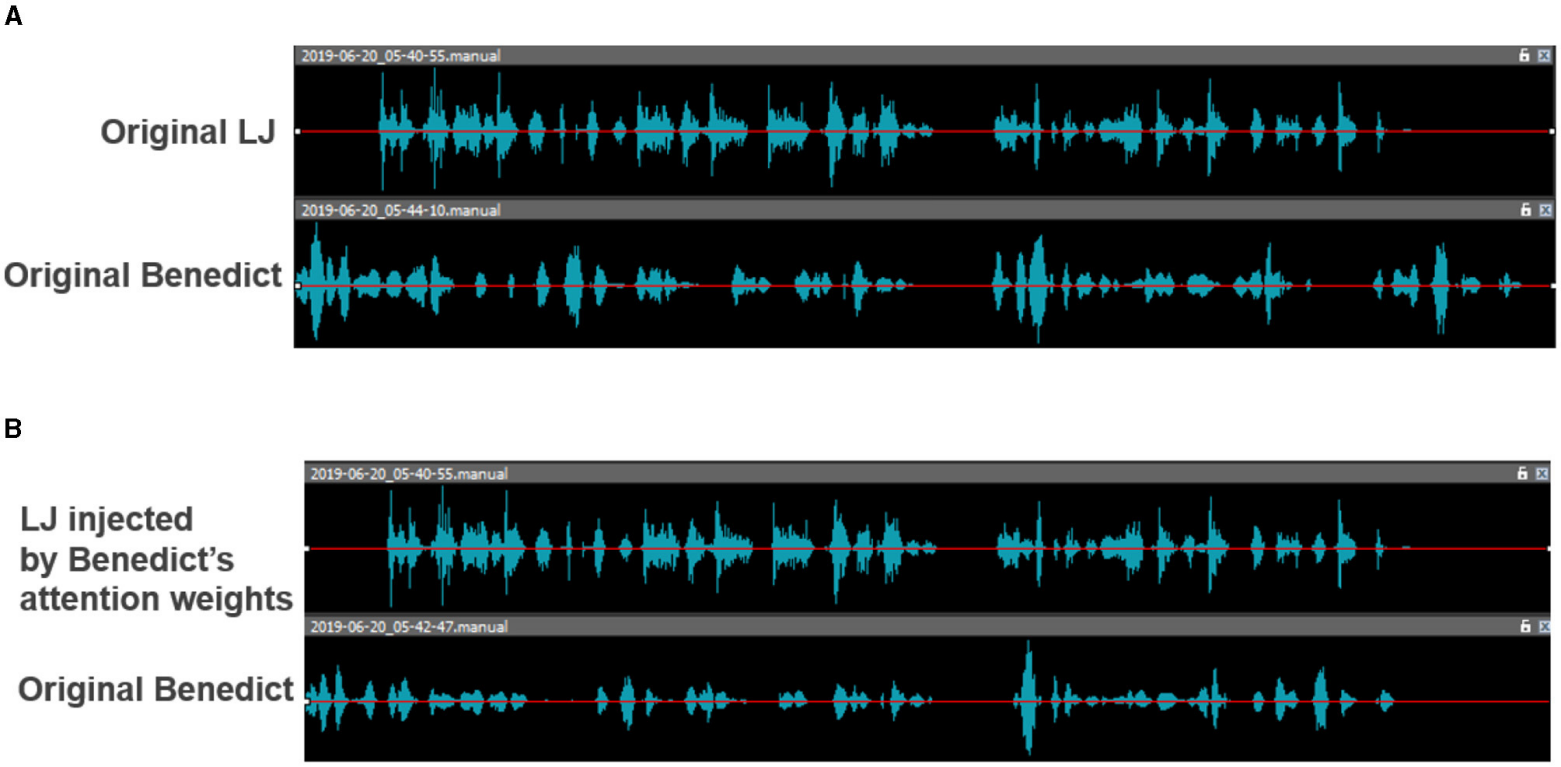
The effects of transferring the learned attention weights to the speech speed. **(A)** Original speeches. **(B)** Transferred speeches.

Figure 2 illustrates the effects of transferring the learned attention weights. In Figure 2A, each speaker speaks at their own speed, while in Figure 2B, their speech speeds are almost the same^4^.

### 3.2 Quantitative analysis

To qualitatively assess the speech similarity with the source speaker achieved by our model in terms of speech intensity, we synthesized three texts for each of the three speakers (Benedict, LJ, and WEB). We then employed five similarity measurement algorithms, namely Euclidean Distance, Manhattan Distance, Cosine Similarity, Jaccard Similarity, and Pearson Correlation Coefficient, to compare the synthesized speech.

To extract the speech features necessary for similarity measurement, we utilized Praat (Kaur and Kaur, 2023), a software tool capable of extracting various speech characteristics. Specifically, at each time interval of 0.001 s, we extracted intensity as shown in Table 2 and converted them to vectors to calculate the speech similarity.

**Table 2:** The example of the extracted intensity feature.

**Time (seconds)**	**0.04925**	**0.053592**	**0.064259**	**0.074925**	**0.085592**	**0.095259**	**0.106925**	**0.117592**	**0.128259**	**0.138925**	**0.149592**
Intensity (dB)	−1.86318	−2.5303	−3.9533	−4.89018	−5.91174	−7.31697	−9.79024	−13.9805	−17.5655	−18.7281	−21.4318

By applying various similarity algorithms, we were able to evaluate the increase in speech similarity resulting from the replacement of the attention module. This analysis provides valuable insights into the effectiveness of our approach in achieving enhanced speech similarity between the target and source speakers. Table 3 shows the actual measurement for a text.

**Table 3:** Similarity analysis between original speeches and the synthesized speeches by our model.

	**Source—target**	**Euclidean distance**	**Manhattan distance**	**Cosine similarity**	**Jaccard similarity**	**Pearson correlation coefficient**
Original	Benedict—LJ	9,439.82	249,387.14	−0.268	0.000635	−0.056
	Benedict—WEB	9,325.08	243,450.65	−0.124	0.000661	0.178
	LJ—WEB	2,872.38	29,209.84	0.937	0.00107	0.894
Proposed	Benedict—LJ with Benedict's attentions	3,328.86	42,275.75	0.628	0.000475	0.613
	Benedict—WEB with Benedict's attenttions	1,845.99	21,343.58	0.852	0.000457	0.838
	LJ—Benedict with LJ's attentions	453.82	6,602.56	0.998	0.00101	0.997
	LJ—WEB with LJ's attentions	379.97	6,511.95	0.999	0.001	0.998
	WEB—Benedict with WEB's attentions	1,354.17	12,251.57	0.986	0.00112	0.974
	WEB—LJ with WEB's attentions	2,484.36	25,282.97	0.953	0.00109	0.917

The analysis of speech similarity for the three texts reveals a consistent and positive trend of increased similarity across all five similarity measurement algorithms. The results can be summarized as follows: (1) Euclidean distance shows an increase of 45.55%, (2) Manhattan distance shows an increase of 55.71%, (3) Cosine similarity shows an increase of 0.3984, (4) Jaccard similarity shows an increase of 4.93%, and (5) Pearson correlation coefficient shows an increase of 0.2957.

These results strongly indicate that the replacement of the attention module with the one from the reference group yields a significant improvement in speech similarity. The observed positive percentage increases across all five similarity measurement algorithms demonstrate the effectiveness of our approach in achieving higher levels of speech similarity between the target and source speakers.

## 4 Discussion and limitations

In this study, we introduced a method to incorporate the source speaker's characteristics into the target speaker, demonstrating its effectiveness. This approach extends beyond the realm of speech synthesis and finds applications in various domains where preserving desired features while leveraging existing models is crucial. Specifically, by extending the attention model, the significant applications can be summarized as follows:

1. Utilizing a trained model with the original speech, we can train an additional model for famous individuals' voices. This enables us to offer narration services, such as audiobooks, using their voices without creating separate narration recordings.
2. The method can be employed in foreign language speech correction services. Not only can we provide static feedback, such as pronunciation correction offered by existing speech-based foreign language correction services, but we can also offer dynamic feedback by analyzing foreign language syllables to achieve more natural speech.
3. Analyzing the voices of voice phishing suspects involved in criminal activities can contribute to crime prevention efforts. By examining and studying the voices of individuals engaged in voice phishing, valuable insights can be gained to enhance prevention strategies.

In this study, we demonstrate that even a very simple approach can cause an effective incorporation of the source speaker's characteristics in the target speaker's speech. However, it has the following limitations, which are orthogonal issues with our solution, to be improved and integrated in future work:

1. We presented our method using the representative end-to-end model, Tacotron. However, our proposed strategy does not depend on a specific model and utilizes general features of voices, which can be extended to other target models.
2. Our study works on speaker-specific models. Because the model needs to be trained for every person, it is limited to extending it to multiple speakers. Although we believe that our experimental settings are efficient to show the effectiveness of the proposed model, our framework can be easily extended to cover more speakers if we can obtain enough training datasets for them.
3. In this study, we mainly utilized the tempo and intensity of the speech focusing on the attention module, showing those simple utilization is clearly effective. We can investigate other speech features such as pitch, formant, pulse, and voice break.
4. Our study focused on the quantitative evaluation to show the effectiveness of the proposed method in an objective way. Therefore, it does not cover the intelligibility or naturalness of the synthesized speech. We can extend it to the qualitative evaluation including subjective listening.

## 5 Conclusions

In this study, we proposed a simple yet effective method for incorporating the source speaker's characteristics in the target speaker's speech, generating the speech of the target speaker with the style of the source speaker. To achieve this, we focused on the attention model within the speech synthesis model, which learns various speaker features. We trained the model separately using datasets specific to the source and target speakers. Subsequently, we replaced the attention weights learned from the source speaker's dataset with the attention weights from the target speaker's model. Finally, by providing new input texts to the target model, we generated the speech of the target speaker with the styles of the source speaker. We validated the effectiveness of our model through similarity analysis utilizing five evaluation metrics and showcase real-world examples.

As future work, we can extend our method to more advanced scenarios such as foreign language learning. In addition, beyond the attention module, we investigate more nuances of speakers' style features. Furthermore, in this study, we focused on the automatic evaluation. However, the evaluation needs to be enhanced including the subject listening tests, intelligibility, and naturalness.

## Data availability statement

Publicly available datasets were analyzed in this study. This data can be found here: https://keithito.com/LJ-Speech-Dataset/; https://www.kaggle.com/datasets/bryanpark/the-world-english-bible-speech-dataset.

## Author contributions

HL: Conceptualization, Data curation, Formal analysis, Investigation, Methodology, Software, Writing – original draft. MC: Conceptualization, Data curation, Formal analysis, Investigation, Methodology, Software, Writing – original draft. H-YK: Conceptualization, Formal analysis, Funding acquisition, Investigation, Methodology, Project administration, Supervision, Validation, Writing – review & editing.

## Footnotes

^1^ https://keithito.com/LJ-Speech-Dataset/

^2^ https://www.kaggle.com/datasets/bryanpark/the-world-english-bible-speech-dataset

^3^The actual demonstration is publicly available: https://drive.google.com/drive/folders/1OQza3e9P73YumLGk6RJVAnkftIcP0E_R.

^4^The actual demonstration is publicly available: https://drive.google.com/drive/folders/1rG5N_4pO0ad78OuRxZskiGAS82Bf54DE.
